# Supplementary material for: Avoiding the Pitfalls of siRNA Delivery to the Retinal Pigment Epithelium with Physiologically Relevant Cell Models
Source: Pharmaceutics. 2020 Jul 16;12(7):667. doi: 10.3390/pharmaceutics12070667 (PMC7407886; doi:10.3390/pharmaceutics12070667)
Supplement: Supplementary file 1 [file pharmaceutics-12-00667-s001.zip › pharmaceutics-852131 - SI formatted/pharmaceutics-852131-SI formatted.pdf]

pharmaceutics

Supplementary Materials: Avoiding the Pitfalls of siRNA Delivery to the Retinal Pigment Epithelium with Physiologically Relevant Cell Models

Eva Ramsay, Manuela Raviña, Sanjay Sarkhel, Sarah Hehir, Neil R. Cameron, Tanja Ilmarinen, Heli Skottman, Jørgen Kjems, Arto Urtti, Marika Ruponen and Astrid Subrizi

PBE_{30}-b-PK_{30}-siRNA polyplexes failed to knockdown IL-6 protein in dividing ARPE-19 cells. Chloroquine treatment did not enhance silencing.

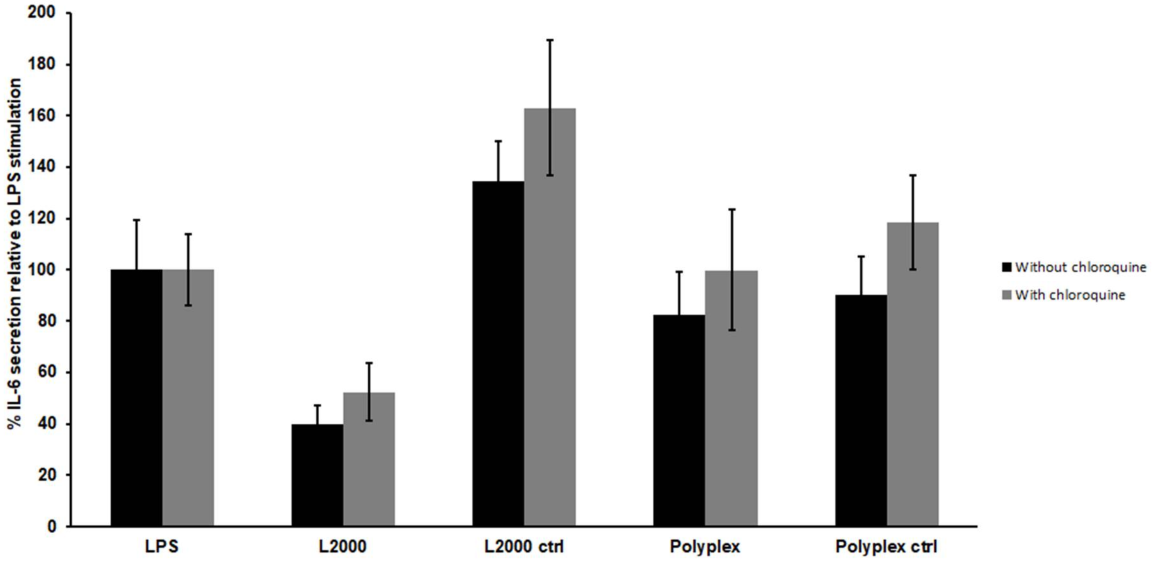

Figure S1. IL-6 knockdown in dividing ARPE-19 cells, 3 days after transfection. Black columns indicate knockdown without chloroquine, and grey columns indicate knockdown with chloroquine treatment. IL-6 protein secretion was evaluated with ELISA and was normalized to LPS treated cells (100%). L2000: Lipofectamine 2000, ctrl: negative control siRNA. Data presented as mean ± SD.
